# Rescuing Nucleus Pulposus Cells From Senescence via Dual‐Functional Greigite Nanozyme to Alleviate Intervertebral Disc Degeneration

**DOI:** 10.1002/advs.202300988

**Published:** 2023-07-03

**Authors:** Yu Shi, Hanwen Li, Dongchuan Chu, Wenzheng Lin, Xinglong Wang, Yin Wu, Ke Li, Huihui Wang, Dandan Li, Zhuobin Xu, Lizeng Gao, Bin Li, Hao Chen

**Affiliations:** ^1^ Department of Orthopedics Affiliated Hospital of Yangzhou University No. 368 Hanjiang Road Yangzhou 225000 P. R. China; ^2^ Institute of Translational Medicine Medical College Yangzhou University No.136 Jiangyang Road Yangzhou 215000 P. R. China; ^3^ Orthopedic Institute Department of Orthopedic Surgery First Affiliated Hospital Suzhou Medical College Soochow University No. 899 Pinghai Road Suzhou 215000 P. R. China; ^4^ Department of Radiology Affiliated Hospital of Yangzhou University No. 368 Hanjiang Road Yangzhou 225000 P. R. China; ^5^ CAS Engineering Laboratory for Nanozyme Institute of Biophysics Chinese Academy of Sciences No. 15 Datun Road Beijing 100101 P. R. China

**Keywords:** cellular senescence, intervertebral disc degeneration, iron sulfide nanoparticle, nanozyme, polysulfides, ROS‐p53‐p21 axis

## Abstract

High levels of reactive oxygen species (ROS) lead to progressive deterioration of mitochondrial function, resulting in tissue degeneration. In this study, ROS accumulation induced nucleus pulposus cells (NPCs) senescence is observed in degenerative human and rat intervertebral disc, suggesting senescence as a new therapeutic target to reverse intervertebral disc degeneration (IVDD). By targeting this, dual‐functional greigite nanozyme is successfully constructed, which shows the ability to release abundant polysulfides and presents strong superoxide dismutase and catalase activities, both of which function to scavenge ROS and maintain the tissue at physical redox level. By significantly lowering the ROS level, greigite nanozyme rescues damaged mitochondrial function in IVDD models both in vitro and in vivo, rescues NPCs from senescence and alleviated the inflammatory response. Furthermore, RNA‐sequencing reveals ROS‐p53‐p21 axis is responsible for cellular senescence‐induced IVDD. Activation of the axis abolishes greigite nanozyme rescued NPCs senescence phenotype, as well as the alleviated inflammatory response to greigite nanozyme, which confirms the role of ROS‐p53‐p21 axis in greigite nanozyme's function to reverse IVDD. In conclusion, this study demonstrates that ROS‐induced NPCs senescence leads to IVDD and the dual‐functional greigite nanozyme holds strong potential to reverse this process, providing a novel strategy for IVDD management.

## Graphical abstract

High levels of reactive oxygen species (ROS) lead to nucleus pulposus cells (NPCs) senescence in degenerative intervertebral disc. A dualfunctional greigite nanozyme is successfully constructed, showing the ability to release abundant polysulfides and presenting strong superoxide dismutase and catalase activities. The greigite nanozyme functions to scavenge ROS and rescue NPCs from senescence, effectively maintaining intervertebral disc homeostasis.

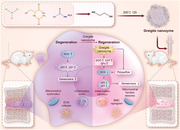

## 1 Introduction

Low back pain (LBP) is a common disorder experienced by humans of all ages. Intervertebral disc degeneration (IVDD) is one of the major factors, easily disabling and seriously affecting life quality.^[^ ^1^, ^2^, ^3^, ^4^ ^]^ Previous studies have observed the senescent nucleus pulposus cells (NPCs) in degenerative human intervertebral discs.^[^ ^5^, ^6^ ^]^ It has been reported that oxidative stress (OS), reactive oxygen species (ROS), and subsequent mitochondrial dysfunction play a key role in the development of cellular senescence.^[^ ^7^, ^8^, ^9^ ^]^ In physiological conditions, ROS mainly concentrates in mitochondria and maintains a dynamic balance.^[^ ^10^ ^]^ When the intervertebral disc is damaged, a large number of ROS, including peroxide (O_2_^•−^), hydrogen peroxide(H_2_O_2_), hydroxyl (•OH), nitric oxide (NO), and singlet oxygen (^1^O_2_), are produced in the closed microenvironment of the intervertebral disc, leading to mitochondrial dysfunction and cellular senescence.^[^ ^11^ ^]^ Senescence is a permanent cell cycle arrest controlled by two major pathways, the p16‐pRb pathway and the p53‐p21 pathway.^[^ ^12^ ^]^ The expression of senescence‐associated *β*‐galactosidase (SA‐*β*‐Gal), p16^INK4*α*^, and p21^Waf1/Cip1^ increases when the NPCs are senescent.^[^ ^13^, ^14^ ^]^ The senescence process is accompanied by decreased anabolic metabolism and increased catabolic metabolism, as well as an inflammatory response, resulting in forming of a malignant microenvironment around NPCs. Therefore, we speculate that the senescence of NPCs can be alleviated by removing excessive ROS, which may improve the functional state of mitochondria, and thus achieve IVDD salvage.

At present, the clinical treatment of IVDD is limited by surgeries only, which are easy to cause severe complications. In recent years, great progress has been made in the research of nano iron sulfides, which has a wide range of applications in biomedical fields such as anti‐microbial, anti‐tumor, biosensing, and drug delivery.^[^ ^15^, ^16^, ^17^, ^18^ ^]^ Previous studies have shown that conventional iron sulfide only exerts antioxidant effects by the release of polysulfides.^[^ ^19^, ^20^, ^21^ ^]^ However, the release of polysulfides from iron sulfide is less efficient. Moreover, the composition of iron sulfide is not homogeneous and is always accompanied by the production of impurities. As a result, there are several properties, including high dose, slow onset of action, and poor controllability, to be improved for conventional iron sulfide. Based on this, we aim to gain the antioxidant activity of iron sulfide which may effectively rescue cell senescence and tissue degeneration caused by oxidative stress and inflammation. Inspired by the dependence of natural enzymes on iron‐sulfur clusters as cofactors acting as active centers for electron transfer in catalytic processes and respiratory chain reactions,^[^ ^22^ ^]^ we tried to synthesize a new type of iron‐sulfides that not only release polysulfides consistently but also mimics the activity of natural antioxidant enzymes. N‐Acetyl l‐Cysteine (NAC), with a sulfhydryl group, is a highly favored and potent non‐specific antioxidant and anti‐inflammatory agent, which is often used in respiratory disorders with high mucus secretion or acetaminophen overdose.^[^ ^23^, ^24^ ^]^ It is concluded that NAC shows good biocompatibility and excellent cytoprotective ability in in vitro oxidative stress microenvironment. Chadwick R. Powell et al.^[^ ^25^ ^]^ applied NAC to synthesize ROS‐responsive per sulfide prodrugs, which effectively alleviated H2O2‐induced oxidative stress damage in H9C2 cardiomyocytes, while this efficacy was demonstrated better than that of Na_2_S, a common donor for H_2_S. Moreover, a recent study of redox‐active thiols found a reduction stress phenomenon, whereby anti‐oxidant cooperation in an oxygenated atmosphere can cause further oxidative stress.^[^ ^26^ ^]^ Based on these evidences, we decided to employ the organosulfur compound NAC as a sulfur source in our study. Basically, we found that iron sulfides release polysulfides (such as H_2_S_2_) in aqueous solutions. It is known that both exogenously and endogenously polysulfides can eliminate intracellular radicals through the process of autocatalytic cycling.^[^ ^27^ ^]^ Polysulfides were found to be ideal H‐atom donors to radicals, including alkyl, alkoxyl, peroxyl, and thiyl.^[^ ^28^ ^]^ This autocatalytic cycle effectively reduces intracellular ROS. Meanwhile, it has been proved that exogenous polysulfides (including H_2_S, H_2_S_2_, etc.) also alleviate oxidative stress by increasing the expression and activities of enzymes, including superoxide dismutase (SOD), catalase (CAT), guaiacol peroxidase (GPX), and so on.^[^ ^29^, ^30^, ^31^ ^]^ Unexpectedly, we observed the newly synthesized iron sulfides possessed remarkable SOD, CAT, and GPx enzymatic activities, which could be related to the antioxidant properties of NAC (the NAC iron sulfide is subsequently named greigite nanozyme). SOD, CAT, and GPx, as natural antioxidant enzymes in living organisms, play important roles in scavenging reactive oxygen species and maintaining normal redox levels in the body.^[^ ^32^ ^]^ In view of the high efficiency of SOD and CAT enzyme activities and polysulfides release functions, we attempted to use greigite nanozyme to remove a large amount of ROS in degenerative intervertebral discs, in order to rebalance the oxidative stress microenvironment.

Therefore, we have carried out a number of studies on the anti‐senescence effect of greigite nanozyme in the process of IVDD from the perspectives of both the oxidoreductase activity of greigite nanozyme and the role of polysulfides release. We discovered that greigite nanozyme or its supernatants reduced oxidative stress level both in vitro and in vivo, improving the mitochondrial function and alleviated NPC senescence. Simultaneously, the process of greigite nanozyme treatment was also accompanied by inhibition of inflammatory response and catabolism, and significantly improved anabolism. Furthermore, through RNA sequencing, we revealed the anti‐senescence effect of greigite nanozyme in NPCs was mediated through regulation of the ROS‐p53‐p21 axis, which was verified by p53 or p21 antagonist. These results suggest that greigite nanozyme mitigate IVDD by inhibiting senescence via scavenging ROS from NPCs, which is regulated through the ROS‐p53‐p21 pathway.

## 2 Results and Discussion

### 2.1 Nucleus Pulposus Cells Senescence Causes Intervertebral Disc Degeneration

**Figure 1:**
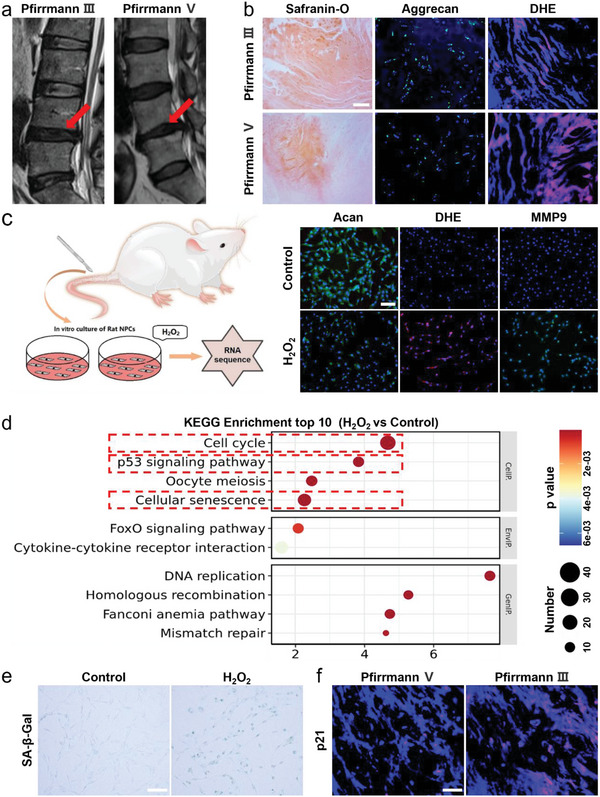
ROS induced nucleus pulposus cell senescence leads to IVDD. a) T2‐weighted MRI scans of human lumbar spine (red arrows indicate the IVDD level). b) Safranin‐O staining of human nucleus pulposus (NP) tissues collected from the cases with of IVDD graded as Pfirrmann III or V (scale bar = 500 µm), Aggrecan (Acan) immunofluorescence and Dihydroethidium (DHE) staining of human NP tissues (scale bar = 100 µm). c) Acan, mmp9 immunofluorescence, and DHE staining of rat NPCs (scale bar = 100 µm). d) KEGG enrichment analysis of the rat NPCs treated with vehicle or H_2_O_2_. e,f) SA‐*β*‐Gal staining and p21 immunofluorescence staining of rat (scale bar = 100 µm).

Lumbar disc degeneration is commonly diagnosed with magnetic resonance imaging (MRI). First, we showed the T2‐weighted MRI scans of two cases with Pfirrmann classification grade III and V, respectively (**Figure** **1**a). Meanwhile, the nucleus pulposus tissue collected from the patients were stained with Safranin‐O, aggrecan (Acan), and Dihydroethidium (DHE). The Safranin‐O and Acan staining results showed the extra cellular matrix (ECM) in the degenerated NP tissue from the Pfirrmann grade V patient was significantly reduced when compared with that in the tissue from the Pfirrmann grade III patient (Figure 1b). DHE staining indicated that the reactive oxygen species (ROS) was increased in the NP tissue from the Pfirrmann grade V patient, showing the reversed trend as to the ECM volume (Figure 1b). The data suggest that the excessive accumulation of intracellular ROS may induce mitochondrial dysfunction, which further reduces the anabolic process and exacerbates the degeneration of nuclear pulposus cells (NPCs).

To further reveal how ROS induces the degeneration of intervertebral disc, we extracted NPCs from the tail intervertebral disc of male SD rats and simulated the cells with H_2_O_2_ to mimic the oxidative stress microenvironment. Immunofluorescent staining of the NPCs showed decreased Acan and increased DHE and mmp9 in the NPCs treated with H_2_O_2_, which successfully imitated the ROS induced NP tissue degeneration in vivo (Figure 1c). Then, RNA sequencing was applied to analyze the transcriptome of NPCs in the control and the H_2_O_2_ treated groups. Kyoto Encyclopedia of Genes and Genomes (KEGG) analysis of the differentially expressed genes between the two groups revealed they mostly enriched in the items of cell cycle, p53 signaling pathway and cellular senescence (Figure 1d). As those signaling pathways are the major ones included in cellular senescence, so we hypothesized that IVDD may be caused by ROS induced senescence of NPCs. Hence, we first confirmed this finding by staining senescence associated‐*β*‐Gal (SA‐*β*‐Gal) in H_2_O_2_ treated cells, which showed significant more senescent NPCs in the H_2_O_2_ treated group as compared to the control (Figure 1e). Furthermore, p21 immunofluorescence staining was performed within the degenerated human intervertebral disc tissue and we observed more p21 expression in the NP tissue from the Pfirrmann grade V patient as to the NP tissue from the Pfirrmann grade III patient (Figure 1f). The above results suggest that ROS induced cellular senescence in NPCs contributes to the degeneration of the intervertebral discs, which provides us a potential therapeutic target to reverse the NP tissue degenerative process.

### 2.2 Physical and Chemical Characterization of Greigite Nanozyme

**Figure 2:**
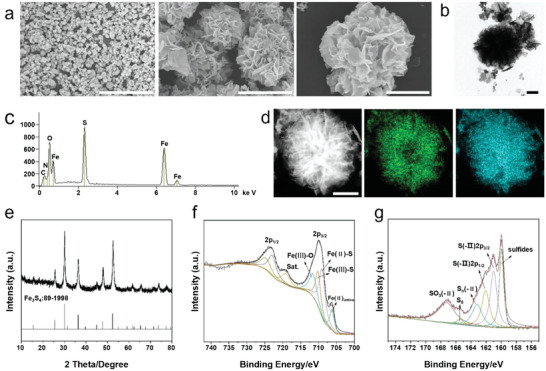
Physical and chemical characterizations of greigite nanozyme. a) SEM image showed the formation of uniformed greigite nanozyme with the appearance of flower‐like microspheres (scale bar = 50 µm, 5 µm, 2 µm). b) TEM images of greigite nanozyme (scale bar = 1 µm). c) EDS of greigite nanozyme confirmed greigite nanozyme contained sulfur (S) and iron (Fe) elements. d) Element mappings of S (green) and Fe (blue) elements in greigite nanozyme. e) The XRD pattern of greigite nanozyme (JCPDS PDF No. 89‐1998). f,g) XPS analysis of the surface chemical composition of the nanozymes.

*N*‐AcNAC‐derived greigite nanozyme was prepared using a simple hydrothermal synthesis method according to the literature.^[^ ^15^ ^]^ SEM showed the formation of uniformed greigite nanozyme with the appearance of flower‐like microspheres (**Figure** **2**a) and each microsphere is mainly formed with lamellar structures. Both SEM and TEM images indicated that the morphology of greigite nanozyme was chrysanthemum‐like with an averaged size of 2 µm (Figure 2b), and the zeta potential of greigite nanozyme was around +12 mV (Figure S1a, Supporting Information). Moreover, in order to observe the degradation process, greigite nanozyme were dissolved in PBS for different time periods and the morphology was observed under SEM (Figure S2, Supporting Information). It showed that the original flower‐like morphology of the greigite nanozyme gradually broke down into small irregular fragments with time. On the 5th day, the greigite nanozyme nanoflowers disintegrated into small fragments, which demonstrated the degradability of the greigite nanozyme. The energy‐dispersive X‐ray spectrum (EDS) suggested that greigite nanozyme mainly consist of sulfur (S) and iron (Fe) (Figure 2c). Element mapping further confirmed that S and Fe elements in the framework of greigite nanozyme with high uniformity (Figure 2d). Furthermore, the crystal structures of greigite nanozyme were characterized by X‐ray powder diffraction (XRD). It is worth mentioning that during the synthesis of greigite nanozyme, we found the challenge of iron oxide impurities. To solve this problem, we utilized different feeds of NAC to synthesize the greigite nanozyme. As shown in Figure S3a–d, Supporting Information, the XRD results showed that the content of iron oxide gradually decreased as the proportion of NAC increased. When the feeding amount of NAC reached 4 mm, the iron oxide almost disappeared and pure Fe_3_S_4_ was synthesized. As shown in Figure 2e, the XRD pattern revealed that the as‐synthesized greigite nanozyme consists of greigite Fe_3_S_4_ (JCPDS PDF No. 89‐1998). Finally, XPS was applied to analyze the surface element composition. The high‐resolution spectra of Fe 2p exhibited four peaks at 706.3, 709, 710.2, and 711.6 eV, which were attributed to Fe (II)_lattice_, Fe (II)‐S, Fe (III)‐S, and Fe (III)‐O, respectively.^[^ ^33^ ^]^ With respect to the S 2p spectra, the peaks at 161 and 162.1 were assigned to S (‐II), and the peaks near 160, 163.2, 165.4, and 167.2 eV corresponded to sulfides, Sn (‐II), S_8_, and SO_3_ (‐II), respectively (Figure 2f,g).^[^ ^34^ ^]^ These results demonstrated the successful construction of greigite nanozyme.

### 2.3 Multiple Enzyme‐Like Activities of Greigite Nanozyme

**Figure 3:**
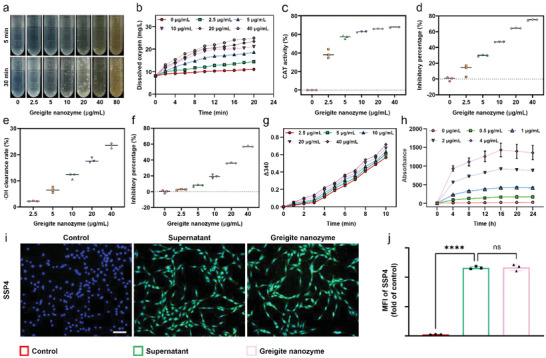
Multiple enzyme‐like activities of greigite nanozyme. a) Photographs of greigite nanozyme (0–80 µg mL^−1^) catalyzing the formation of oxygen bubbles in H_2_O_2_ (800 mm) solution after incubation for 5 min and 30 min. b) Measurement of oxygen release from H_2_O_2_ (800 mm) catalyzed by greigite nanozyme. c) CAT‐like activity of greigite nanozyme. d) SOD‐like activity and ^•^O_2_^•−^ scavenging capacity of greigite nanozyme. e) ^•^OH scavenging capacity of greigite nanozyme. f) Determination of total antioxidant capacity of greigite nanozyme. g) Detection of glutathione peroxidase‐like activity of greigite nanozyme. h) The release trend of polysulfides from greigite nanozyme. i,j) The release of polysulfides from greigite nanozyme in NPCs and semi‐quantitative analysis of the release amount (scale bar, 100 µm). ****p < 0.0001.

The development of IVDD is accompanied by excessive accumulation of ROS, the essential mediators of inflammation, senescence and apoptosis.^[^ ^35^, ^36^, ^37^, ^38^, ^39^, ^40^ ^]^ Greigite nanozyme may protect cells from oxidative stress by scavenging varieties of ROS, including H_2_O_2_, O_2_^•−^, ^•^OH, etc. First, the catalase (CAT) like activity of greigite nanozyme was evaluated by directly detecting the formation of O_2_ bubbles. As shown in **Figure** **3**a, adding of greigite nanozyme into H_2_O_2_ solution quickly catalyzed to form O_2_ bubbles under 37 °C. The production of O_2_ gradually increase with the elevated concentrations of greigite nanozyme and the extension of time. In addition, the O_2_ content from H_2_O_2_ catalyzed by greigite nanozyme within 20 min at 37 °C was monitored using dissolved oxygen meter (Figure 3b). Greigite nanozyme (20 µg mL^−1^) catalyzed to release O_2_ from H_2_O_2_ in 20 min up to 23.39 mg L^−1^, which was approximately more than twice the amount of O_2_ (11.02 mg L^−1^) as compared with no greigite nanozyme. The catalytic efficiency of greigite nanozyme in catalyzing H_2_O_2_ to O_2_ within 5 min was shown in Figure 3c, and the 5‐min catalytic efficiency of greigite nanozyme in 20 µg mL^−1^ reached 65.99%. Meanwhile, SOD is a classical antioxidant enzyme that converts O_2_^•−^ into H_2_O_2_ and O_2_.^[^ ^41^ ^]^ The SOD‐like activity of greigite nanozyme was investigated by using the superoxide dismutase kit. The results showed that, when the concentration of greigite nanozyme grew, the inhibition rate increases prominently 20 min after the addition of the reaction reagent (Figure 3d). In addition, the quasi‐peroxidase (POD) activity and quasi‐oxidase (OXD) activity of greigite nanozyme were determined at pH 4.5 and pH 6.5. As shown in Figure S4a–d, Supporting Information, greigite nanozyme has weak POD activity and OXD activity, which could hardly convert H_2_O_2_ into toxic free radicals in the acidic environment.^[^ ^42^, ^43^, ^44^ ^]^ However, greigite nanozyme showed a limited ability to remove hydroxyl radical (^•^OH), another reactive oxygen species (Figure 3e).

The amount of antioxidant macromolecules, antioxidant small molecules, and enzymes are considered to represent the levels of total antioxidant capacity. Hence, the total antioxidant capacity of greigite nanozyme was evaluated, showing its ideal antioxidant capacity, which was positively correlated with the greigite nanozyme concentration (Figure 3f). In addition, the intracellular lipids react with free radicals to produce lipid peroxides, which ultimately leads to oxidative damage and cell death. Glutathione peroxidase (GPx) plays an important role in maintaining ROS metabolic homeostasis by catalyzing GSH to GSSG through eliminating the free radicals. Our results showed that greigite nanozyme also exhibited good GPx‐like activity, reducing lipid peroxides effectively in a dose‐ and time‐dependent manner (Figure 3g).

We next verified the release of polysulfides from the greigite nanozyme with the Sulfane Sulfur Probe 4 (SSP4) kit. SSP4 is non‐fluorescent but emits strong green fluorescence when reacts with sulfane sulfurs. Different concentrations of greigite nanozyme were dissolved in PBS and the release trend of polysulfides were determined at different time points by using SSP4 kit. As shown in Figure 3h, polysulfides were released steadily and continuously over time and reached a plateau at around 16 h. Moreover, we used greigite nanozyme and its supernatant to test the release of polysulfides within NPCs separately. The results showed that the green fluorescence increased in the NPCs in both the supernatant and greigite nanozyme stimulating groups, suggesting the release of polysulfides from the greigite nanozyme, and no difference was observed between these two groups, indicating the amount of polysulfides that release was definite (Figure 3i,j). To sum up, the polysulfides release capacity, multiple enzyme‐like activities, and the prominent ROS scavenging capacity of greigite nanozyme were well characterized, which were expected to alleviate the troublesome oxidative stress microenvironment in degenerative disc disorders and serve as a promising antioxidant for IVDD management.

### 2.4 Greigite Nanozyme Repairs Damaged Mitochondria by Removing Excessive ROS

We isolated NPCs from SD rats and identified the cells with toluidine blue and Aggrecan staining.^[^ ^45^ ^]^ The toluidine blue staining showed the accumulation of proteoglycans, stained as indigo blue, in the NPCs isolated (Figure S5a, Supporting Information), which showed the extensive expression of aggrecan in the cells as well (Figure S5b, Supporting Information). The results confirmed the cells isolated from rat tail discs are NPCs.

**Figure 4:**
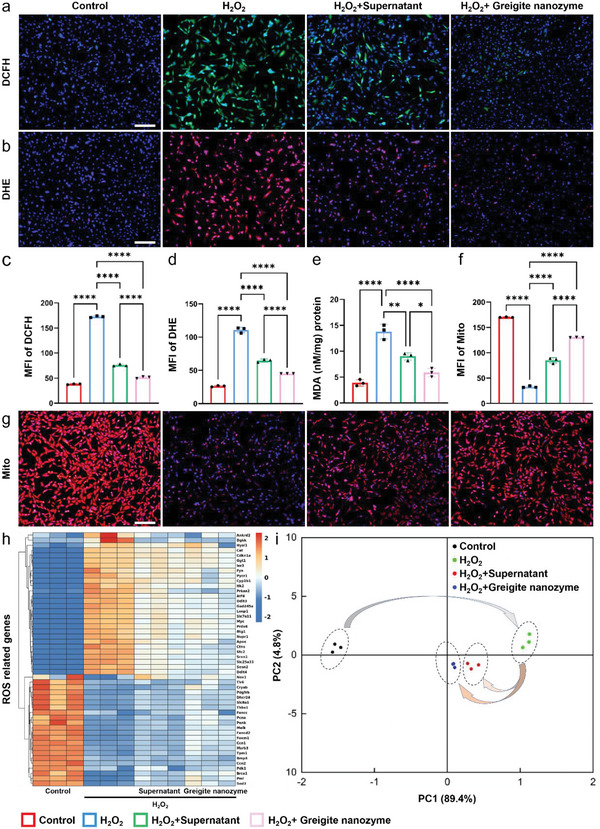
Greigite nanozyme repairs damaged mitochondria by removing excessive ROS. a,b) DCFH and DHE fluorescent staining images show ROS levels in NPCs (scale bar = 100 µm). c,d) Semi‐quantitative analysis of DCFH and DHE staining. e) The expression level of malondialdehyde in NPCs. f,g) Mitochondrial membrane potential staining of NPCs and the semi‐quantitative analysis of fluorescence intensity (scale bar = 100 µm). h) Unsupervised clustering of the ROS related genes in the control, H_2_O_2_ treated, H_2_O_2_+Supernatant and H_2_O_2_+ greigite nanozyme groups (*n* = 3). i) Principal component analysis of the relationship among the four groups (*n* = 3) (*p < 0.05; **p < 0.01; ****p < 0.0001).

H_2_O_2_ has been commonly considered as a natural inducer of oxidative stress, which could contribute to mitochondrial dysfunction cell senescence.^[^ ^46^ ^]^ In order to identify the oxidative stress microenvironment of NPCs, the cell viability of NPCs treated with different concentrations of H_2_O_2_ was tested using the cell counting kit 8 (CCK8) assay. With the concentration of H_2_O_2_ increased to 100 µm, the cell viability decreased to 61.7 ± 3.3% compared to the control group (Figure S6c, Supporting Information). Likewise, Calcein/PI staining showed the increase of H_2_O_2_ concentration gradually induced cell death (Figure S6a,b, Supporting Information). In view of the clear sublethal cytotoxicity of 100 µm hydrogen peroxide, this concentration was used to simulate oxidative stress microenvironment in NPCs. On top of that, the cell viability was tested using CCK8 assay under gradient concentrations of greigite nanozyme incubated together for 24 h. The ideal concentration of greigite nanozyme was 1 µg mL^−1^, which was chosen as the concentration for the following tests (Figure S6d, Supporting Information). Then, we tested if the greigite nanozyme could rescue the degenerated NPCs from the oxidative stress induced by H_2_O_2_. The NPCs were pre‐incubated with different concentrations of greigite nanozyme for 1 h and then added with 100 µm H_2_O_2_ for 24 h. The results showed 1 µg mL^−1^ greigite nanozyme exerted the best inhibitory function on H_2_O_2_ induced oxidative stress in NPCs (Figure S6e, Supporting Information). Since the dual function of greigite nanozyme as redox enzyme activity and polysulfides release, we performed the following experiments with greigite nanozyme and its supernatant, respectively. First, we used DCFH‐DA and DHE probes to detect the ROS level in NPCs. As shown in **Figure** **4**a,b, when NPCs were exposed to H_2_O_2_, the ROS expression level increased sharply, accompanied by the change of cell morphology from fusiform to irregularly round. Then, the ROS level reduced significantly when the supernatant or greigite nanozyme was added, in which the ROS scavenging efficiency was stronger in the greigite nanozyme group than the supernatant group. The fluorescence quantification results were showed in Figure 4c,d. Malondialdehyde (MDA) is the natural product of lipid oxidation in organism. In oxidative stress microenvironment, lipid oxidation occurs in NPCs, resulting in MDA production. The supernatant showed satisfactory inhibition effect on MDA production, which was even better in the greigite nanozyme group (Figure 4e). The main ROS production site in non‐immune cells is mitochondria.^[^ ^47^ ^]^ The accumulation of intracellular ROS can easily cause oxidative damage to mitochondria, while ROS‐induced mitochondrial dysfunction further enhances the production of ROS, forming a vicious cycle that leads to continuous intracellular oxidative damage.^[^ ^8^ ^]^ Therefore, the clearance of intracellular ROS has a close relation with mitochondrial state regulation. To test the function of mitochondria in nucleus pulposus cells, Mito‐Tracker Red CMXRos was applied. The results of mitochondrial fluorescence staining and the quantitative analysis of fluorescence intensity was shown in Figure 4f,g. H_2_O_2_ treatment decreased the fluorescence expression level in NPCs, indicating the damage of the mitochondria. Then, application of the supernatant and greigite nanozyme alleviated the mitochondrial damage of NPCs to different degrees. Furthermore, as reflected by the RNA‐seq data, heat map analysis and principal component analysis (PCA) with ROS‐related genes showed significant difference between the H_2_O_2_ treated group and the control group, while the difference was greatly reversed under the effect of the supernatant or greigite nanozyme (Figure 4 h,i). Similar to the staining results, the ROS genes expression pattern was closer to the control group while the greigite nanozyme was added as compared to the supernatant group no matter reflected by the heatmap of the PCA. The results show the prominent capacity to repair dysfunctional mitochondrial by eliminating excess ROS due to the dual functions of greigite nanozyme.

### 2.5 Greigite Nanozyme Inhibits NPC Senescence and Rescues Damaged NPCs under Oxidative Stress Microenvironment

**Figure 5:**
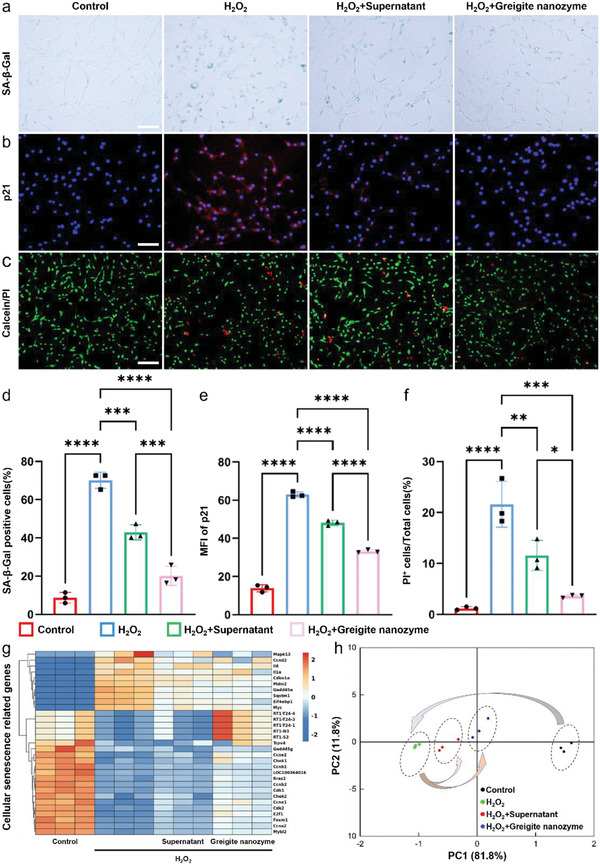
Greigite nanozyme rescues NPCs from senescence in oxidative stress microenvironment. a) Senescence‐associated *β*‐galactosidase staining of NPCs in different groups (scale bar = 100 µm). b) p21 immunofluorescence staining of NPCs in different groups (scale bar = 100 µm). c) The calcein/PI staining of NPCs cultured by greigite nanozyme or its supernatant with H_2_O_2_ (scale bar = 200 µm). d) Semi‐quantitative analysis of the expression level of SA‐*β*‐Gal positive cells in in different groups. e) Semi‐quantitative analysis of p21 fluorescence intensity in different groups. f) Semi‐quantitative analysis of Calcein/PI fluorescence intensity in different groups. g,h) Heatmap of genes enriched in cellular senescence pathway and results of principal component analysis in different groups (*n* = 3, log_2_FC > 1, *p*‐value < 0.05) (* means *p*‐value < 0.05, ** means *p*‐value < 0.01, *** means *p*‐value < 0.001, **** means *p*‐value < 0.0001).

Mitochondrial ROS have been shown to induce DNA damage and accelerate telomere‐induced senescence.^[^ ^48^ ^]^ When telomeres are exposed to oxidative stress microenvironment, they have been shown to cause single strand breaks, and the accumulation of broken single strands leads to accelerated telomere shortening, thus exacerbating cellular aging.^[^ ^49^ ^]^ Accordingly, intervention on mitochondria to remove ROS in mitochondria has been proved to slow down the rate of telomere shortening and thus alleviate cell senescence.^[^ ^50^, ^51^ ^]^ Andria R. Robinson et al.^[^ ^52^ ^]^ demonstrated mitochondrial targeted free radical scavenger therapy delays the occurrence of age‐related IVDD. In this study, we observed the ROS accumulation induced cellular senescence in human and rat degenerative intervertebral discs. We then carried out senescence related staining to further explore the dual function of greigite nanozyme in fighting against IVDD. It showed increased SA‐*β*‐Gal and p21^Waf1/Cip1^ staining in NPCs with H_2_O_2_ induced excessive oxidative stress (**Figure** **5**a,b), while the supernatant and greigite nanozyme suppressed their expression efficiently, in which the greigite nanozyme group was even lower. In addition, greigite nanozyme and its supernatant significantly rescued the NPCs from death under H_2_O_2_ induced oxidative stress conditions. However, the rescuing effect by greigite nanozyme was stronger than its supernatant (Figure 5c). Semi‐quantitative analysis of SA‐*β*‐Gal, p21, and Calcein/PI staining were shown in Figure 5d–f. Heat map and PCA showed a remarkable difference in cellular senescence related genes in the H_2_O_2_ group compared to the control group, while the difference was partly reversed when the supernatant or greigite nanozyme was added, in which the greigite nanozyme functioned more effectively (Figure 5 g,h). Similarly, the greigite nanozyme group had a better inhibitory effect on cellular senescence than the supernatant group. These results demonstrated that greigite nanozyme with dual function as multiple enzyme‐like activities and the release of polysulfides have significant anti‐senescence effects.

### 2.6 Greigite Nanozyme Re‐Establishes the Anabolic and Catabolic Balance to Maintain NPCs Homeostasis

**Figure 6:**
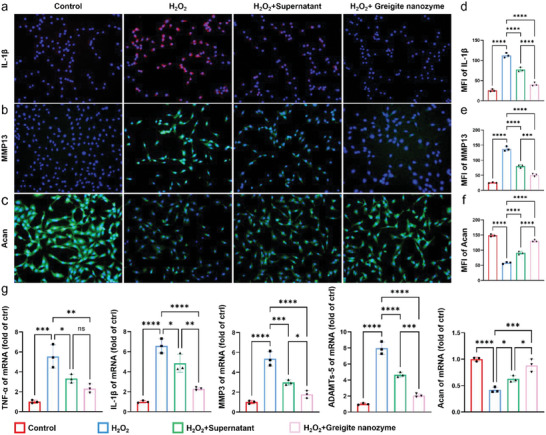
Greigite nanozyme re‐establishes the anabolic and catabolic balance to maintain NPCs homeostasis. a–c) Immunofluorescence staining results of IL‐1*β*, MMP13, and Acan in different groups (scale bar = 100 µm). d–f) Semi‐quantitative analysis of fluorescence intensity of IL‐1*β*, MMP13, and Acan. g) The mRNA expression levels of TNF‐*α*, IL‐1*β*, MMP3, ADAMTs‐5, and Acan in different groups of NPCs (* means *p*‐value < 0.05, ** means *p*‐value < 0.01, *** means *p*‐value < 0.001, **** means *p*‐value < 0.0001).

The process of oxidative stress induced senescence is always accompanied by secretion of pro‐inflammatory cytokines, extra cellular matrix degradation proteins and chemokines, which are collectively referred to as senescence associated secretory phenotypes (SASP).^[^ ^53^ ^]^ After H_2_O_2_ stimulation, the expression of pro‐inflammatory cytokines (such as TNF‐*α* and IL‐1*β*) and catabolic related proteins (such as MMP3, MMP9, MMP13, and ADAMTs‐5) were significantly increased in NPCs, accompanied with the decreased anabolic related proteins (such as Aggrecan and Collagen II) expression (**Figure** **6**a–f; Figure S7a–f, Supporting Information), indicating the disruption of the homeostasis within the NPCs. The change of this complex metabolic process was also an important aspect of IVDD pathology.^[^ ^54^ ^]^ Then, we observed the function of both supernatant and greigite nanozyme to rebalance the situation, with stronger effect by the greigite nanozyme. Similar results were further confirmed by qPCR analysis in the RNA level (Figure 6g). The above data reveal the capacity of greigite nanozyme in controlling ROS induced micro‐environment disorders within the degenerative intervertebral discs.

### 2.7 Greigite Nanozyme Rescues NPCs from Senescence through p53‐p21 Signaling Pathway

**Figure 7:**
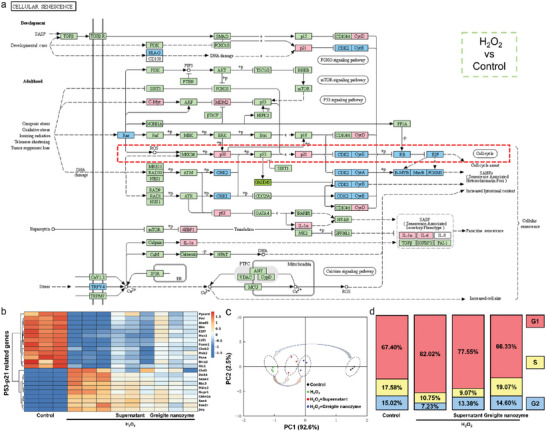
ROS induces NPC senescence through p53‐p21 signaling pathway. a) KEGG‐map of cell senescence signal pathway from group of H_2_O_2_ versus Control. Red box indicates the ROS‐p53‐p21 axis within the cellular senescence pathway. b) Unsupervised clustering of the genes in p53‐p21 signaling in the control, H_2_O_2_ treated, H_2_O_2_+Supernatant and H_2_O_2_+ greigite nanozyme groups (*n* = 3). c) Principal component analysis of the relationship among the four groups (*n* = 3). d) The proportion of NPCs in G1, S, and G2 cell cycle phase in the control, H_2_O_2_ treated, H_2_O_2_+Supernatant, and H_2_O_2_+greigite nanozyme groups.

As we have confirmed the cellular senescence rescuing ability of the greigite nanozyme on NPCs, nevertheless, how it works is still unclear. Based on the transcriptome data, we found that among the highly ranked pathways in KEGG enrichment analysis, the cell cycle, p53 signaling pathway and cellular senescence pathway showed remarkable differences. In addition, as shown in **Figure** **7**a, in the KEGG map of cellular senescence, ROS‐p53‐p21‐cell cycle pathway was activated.

**Figure 8:**
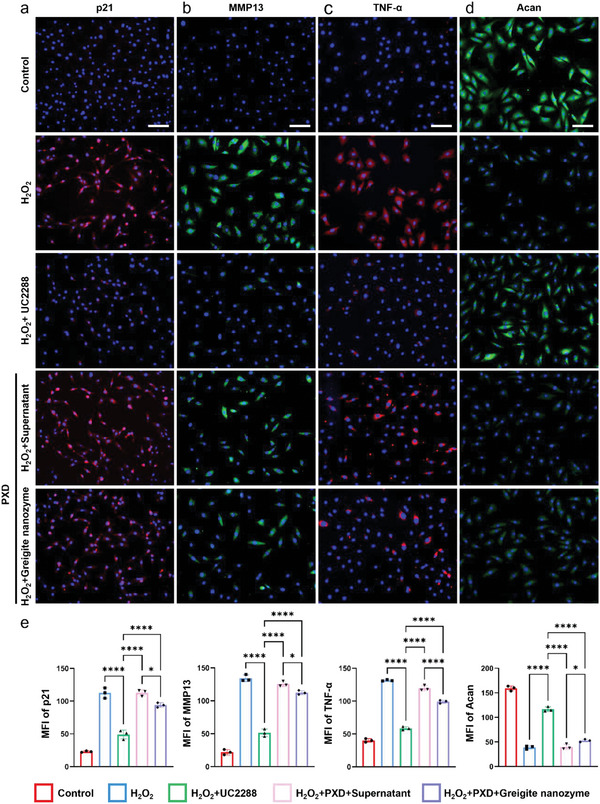
Activation of p21 abolishes NPC senescence rescuing phenotype. Immunofluorescence staining of a) p21, b) MMP13, c) TNF‐*α*, and d) Acan of NPCs from the groups including Control, H_2_O_2_, H_2_O_2_+UC2288, H_2_O_2_+Supernatant+Phenoxodiol (PXD), and H_2_O_2_+greigite nanozyme+PXD (scale bar = 100 µm). e) Semi‐quantitative analysis of fluorescence intensity of p21, MMP13, TNF‐*α*, and Acan (* means *p*‐value < 0.05, **** means *p*‐value < 0.0001).

Hence, we first mapped the p53‐p21 signaling pathway‐regulated cell cycle arrest should be responsible for H2O2‐induced cellular senescence. Heat map and PCA with P53‐P21 related genes expression pattern in the H_2_O_2_ group and the control group was totally different, which was reversed to various degrees by the supernatant or greigite nanozyme (Figure 7b,c). Furthermore, cell cycle analysis showed NPCs treated with H_2_O_2_ displayed a shift toward the G1 phase, which was reversed by the supernatant or greigite nanozyme (Figure 7d). According to research, cell cycle transition to G1 phase is inextricably linked to cellular senescence.^[^ ^55^, ^56^ ^]^ Therefore, it was reasonable to speculate that greigite nanozyme may inhibit oxidative stress‐induced senescence of NPCs by activating the p53‐p21 signaling pathway. Based on the above results, we focused on the p53‐p21 signaling pathway. Phenoxodiol (PXD) and UC2288, known as p21 agonists and inhibitors, are commonly used in aging and cell cycle studies.^[^ ^57^, ^58^, ^59^ ^]^ Then, the PXD (agonist) or UC2288 (antagonist) was added to the H2O2‐treated NPCs simultaneously with greigite nanozyme or its supernatant. From the results of immunofluorescent staining, the NPC senescence induced by H_2_O_2_ was obviously down‐regulated by UC2288 along with the inhibited expression of p21. On the contrary, when PXD was added to the H2O2‐treated NPCs, the senescence‐rescuing effect by greigite nanozyme or its supernatant was mostly abolished (**Figure** **8**a). In parallel, we also performed validation of the catabolic factors, and a similar trend was obtained for immunofluorescence staining of mmp13 and TNF‐*α* (Figure 8b,c). For the anabolic reaction, an opposite trend was observed as to the p21 and the catabolic factors (Figure 8d). The semi‐quantitative analysis of the fluorescence intensity of the above staining was shown in Figure 8e. From the above experimental results, it can be concluded that greigite nanozyme inhibits the NPC senescence by blocking the p21 signaling pathway.

### 2.8 Local Injection of Greigite Nanozyme Reverses IVDD In Vivo

**Figure 9:**
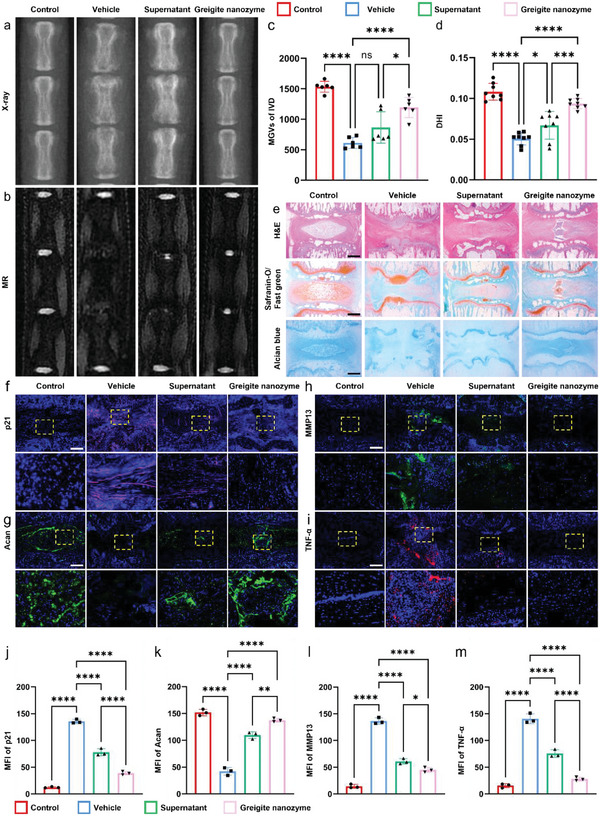
Local injection of greigite nanozyme reverses IVDD in vivo. a,b) X‐ray and MRI images of rat tail from the sham group, puncture group, puncture with greigite nanozyme supernatant (0.2 µg) injection group, and puncture with greigite nanozyme (0.2 µg) injection group. Rats were sacrificed and the samples were collected 4 weeks after injection. c) Mean gray value of the target intervertebral disc (IVD). d) DHI of rat caudal IVD. e) Hematoxylin/eosin, safranin‐O/fast green, and alcian blue staining of the rat caudal IVD (scale bar = 1000 µm). f) p21, g) Acan, h) MMP13, i) TNF‐*α* immunofluorescence staining of the rat caudal IVD sections from the above four groups (scale bar = 400 µm). Semi‐quantitative analysis of fluorescence intensity of j) p21, k) Acan, l) MMP13, m) TNF‐*α* (* means *p*‐value < 0.05, **** means *p*‐value < 0.0001).

We successfully established IVDD model in vivo by needling the vertebral disc of SD rats. After puncture, the supernatant or greigite nanozyme (20 µg mL^−1^, 10 µL) was injected into the intervertebral disc immediately, and equal volume PBS solution was injected into the control model group. The degree of IVDD was measured by MRI and X‐ray at 4 weeks after surgery. The results showed the most severe IVDD in the puncture model group, which was partly reversed by the supernatant or greigite nanozyme as reflected by increased intervertebral disc water content, enhanced T2‐weighted signal, recovery of intervertebral disc height, and decreased osteophytes in the intervertebral space (**Figure** **9**a–c). As expected, the greigite nanozyme group performed even better than that of the supernatant group. The variation of disc height is another criterion to reflect the degeneration of IVD, which is referred as disc height index (DHI). We observed a significant improvement of DHI in the supernatant and greigite nanozyme groups compared to the IVDD group (Figure 9d). In addition to radiological results, histomorphological staining also showed significant differences among these groups. In the puncture group, the intervertebral space collapsed and the intervertebral disc underwent severe fibrotic change. The upper and lower endplates, as well as the growth plate, were also destroyed to a certain extent. In the supernatant and greigite nanozyme groups, the nucleus pulposus, annulus fibrosus, and the endplates were intact as revealed by H&E, safranin‐O, and alcian blue staining (Figure 9e). Besides, the expression of p21 in the degenerative intervertebral disc model was significantly inhibited with the supernatant or greigite nanozyme injected (Figure 9f). In accordance with the in vitro data, immunofluorescence staining revealed that the supernatant or greigite nanozyme enhanced the anabolism and inhibited the catabolism in the nucleus pulposus tissue with Aggrecan expression increased and MMP13, TNF‐*α* expression decreased (Figure 9g–i). These results suggest that both the supernatant and greigite nanozyme attenuated needled‐induced intervertebral disc degeneration through reduction of the oxidative stress level induced cellular senescent in the nucleus pulposus.

## 3 Discussion

In this work, we demonstrate that the greigite nanozyme (i.e., pure Fe_3_S_4_) is an effective candidate for delaying disc degeneration, which effectively improves the conditions of malignant oxidative stress within the NPCs microenvironment in a bifunctional manner, that is, inhibits the senescence of NPCs induced by ROS in a synergistic manner by releasing polysulfides and exerting antioxidant enzyme activity. In particular, the greigite nanozyme showed a great potential for IVDD treatment for those challenged by the indecisive selection of conservative treatment or traumatic surgical procedure.

In recent years, research into nanomaterials in the field of IVDD has been in full swing. For instance, Jie Hao et al. chemically grafted manganese dioxide (MnO_2_)‐lactate oxidase (LOX) composite nanozyme onto microfluidic hyaluronan methacrylate (HAMA) microspheres and realized effective repair of IVDD induced by lactic acid.^[^ ^60^ ^]^ In addition, Prussian blue nanoparticles stabilized SOD1 from ubiquitination‐proteasome degradation to rescue IVDD.^[^ ^61^ ^]^ However, there appear to be only a few applications for iron sulfide nanoparticles in IVDD. Previous studies have reported that iron sulfides release polysulfides and exerts excellent antibacterial properties.^[^ ^15^, ^62^, ^63^, ^64^, ^65^ ^]^ In addition, polysulfides have also been proved to involve in the field of antiviral therapy. Unlike conventional antiviral approaches, the polysulfides released by the broad‐spectrum antiviral metastable iron sulfides (mFeS) inhibit intracellular viral replication through inhibition of lipid peroxidation and ferroptosis in cells, thus enabling effective treatment of influenza viruses.^[^ ^19^ ^]^ Other studies have also demonstrated that exogenously supplied and endogenously produced polysulfides significantly scavenge intracellular free radicals, which is derived from the autocatalytic cycle of polysulfides and the interconversion between GSH and GSSH.^[^ ^27^, ^66^ ^]^ Notably, polysulfides hold the ability to rapidly penetrate cell membranes and thus exert their antioxidant effects intracellularly in a time manner.^[^ ^67^ ^]^ In the oxidative stress microenvironment that the degenerative NPCs exposed to, greigite nanozyme is thus more likely to contact and scavenge ROS through the autocatalytic cycle of polysulfides, as the polysulfides effectively terminate free radical chain reactions by generation and recombination of perthiyl radicals. The release of polysulfides thus provides new intellection for the therapeutic application of iron sulfide in biomedicine. In our study, NAC was employed as sulfur source for greigite nanozyme synthesis. Unexpectedly, the greigite nanozyme showed strong SOD, CAT, and GPx enzymatic activities in addition to the polysulfides property. However, the remarkable antioxidant‐like enzymatic activity of greigite nanozyme does not seem to be able to stand apart from NAC. NAC itself is a stable antioxidant and sulfur source donor.^[^ ^26^ ^]^ In recent years, NAC showed extensive and far‐reaching potential in the treatment of ROS‐related kidney injury, myocardial injury, diabetes‐related wound healing and so on.^[^ ^68^, ^69^, ^70^, ^71^, ^72^ ^]^ In addition, NAC appears as a raw material in the synthesis of nanomaterials for a wide application, which has dabbled in the fields of drug delivery, medical imaging and so on.^[^ ^73^, ^74^, ^75^ ^]^

In the microenvironment of intervertebral disc degeneration, the accumulation of ROS triggers a series of problems including mitochondrial dysfunction, NPC senescence, and the continued secretion of SASP.^[^ ^76^ ^]^ It is worth mentioning that the greigite nanozyme we synthesized significantly inhibited ROS at a low concentration of 1 µg mL^−1^. This concentration is much lower than previously reported application of iron sulfides, which we think could be related to the synergistic antioxidant effect of the released polysulfides and the enzyme activities from the greigite nanozyme. Moreover, the flower‐like greigite nanozyme synthesized in this study provides a higher surface‐to‐volume ratio in comparison with spherical nanoparticles, which facilitates the efficiency of surface reactions.^[^ ^77^ ^]^ In the oxidative stress microenvironment, the larger relative surface area of the material is able to provide more contact area for ROS, which may help to exert the antioxidant enzyme activity of greigite nanozyme more efficiently. In addition, compared with lamellar structure, the flower‐like morphology is more stable, which is conducive to the continuous development of antioxidant activity. Furthermore, the flower‐like morphology feature also possesses other potential applications, such as dye adsorption, enzyme immobilization, etc.^[^ ^78^ ^]^

As shown in the experimental results, distinct from the inherent peroxidase (POD)‐like activity of some iron nanomaterials,^[^ ^79^ ^]^ greigite nanozyme (Fe_3_S_4_) exhibited negligible POD‐like and OXD‐like enzymatic activity in an acidic environment. Therefore, greigite nanozyme showed satisfactory biocompatibility under acidic microenvironment. More importantly, impressive ROS scavenging capacity of greigite nanozyme was still remained under pH 6.5. The extraordinary antioxidant activity of greigite nanozyme in vitro and in vivo was dependent on the synergistic effect of sustained release of polysulfides and the antioxidant enzyme activity, which then rescued the damaged mitochondria from malfunctions and subsequently reversed the IVDD. We have creatively applied this unique bifunctional greigite nanozyme to the model of IVDD and effectively alleviated disc degeneration, providing a good application scenario. In conclusion, in the present study, we investigated the dual functions of greigite nanozyme to alleviate IVDD through inhibiting ROS‐induced NPC senescence, and it was further suggested that the protective mechanism of greigite nanozyme relies on the inhibition of p53‐p21 signaling pathway. Take together, the above results provide potential targets for the prevention and management of IVDD.

## 4 Experimental Section

### Reagents and Antibodies

Collagenase type II, Matrix Metalloproteinase‐13 (mmp13), Goat Anti‐Mouse IgG H&L, and Goat Anti‐Rabbit IgG H&L were purchased from Abcam (China). Aggrecan was purchased from Abclonal (China). Matrix Metalloproteinase‐9 (mmp9), TNF‐*α* were purchased from Proteintech (China). IL‐1*β* and p21^Waf1/Cip1^ were purchased from Santa Cruz Biotechnology (China). Hydrogen peroxide (H_2_O_2_), sodium acetate trihydrate (NaOAc), pentobarbital sodium, and ethylene glycol (EG) were purchased from Aladdin (China). Ferric trichloride hexahydrate and Safranin‐O fast green were purchased from Sigma (China). Dulbecco's Modified Eagle Medium/Nutrient Mixture F‐12 (DMEM/F‐12) and Fetal bovine serum (FBS) were purchased from Gibco (America). The superoxide dismutase (SOD) kit was purchased from Cominbio (China). SSP4 (Sulfane Sulfur Probe 4) fluorescein was purchased from Dojindo (Japan). Total Antioxidant Capacity Assay Kit, Total Glutathione Peroxidase Assay Kit, Calcein/PI Cell Viability/Cytotoxicity Assay Kit, Mito‐Tracker Red CMXRos, Cell Cycle and Apoptosis Analysis Kit, Cell Counting Kit‐8, Cell lysis buffer for Western and IP, Antifade Mounting Medium, Dimethyl Sulfoxide, Quick Antigen Retrieval Solution for Frozen Sections, Alcian Blue Staining Kit, Reactive Oxygen Species Assay Kit, Dihydroethidium, Lipid Peroxidation MDA Assay Kit, and Hematoxylin and Eosin Staining Kit were purchased from Beyotime (China). Trypsin‐EDTA solution, phosphate buffered solution, hydroxyl radical scavenging ability detection kit, Bovine Serum Albumin, Senescence *β*‐Galactosidase Staining Kit, Antibody Diluent, EDTA decalcifying solution DAPI Dihydrochloride, Toluidine Blue Cartilage Stain Solution, 4% Paraformaldehyde were purchased from Solarbio (China).

### Preparation of Greigite Nanozyme

Greigite nanozyme was synthesized with hydrothermal synthesis method. First, 0.82 g FeCl_3_•6H_2_O was added into 40 mL ethylene glycol, stirring at room temperature for 30 min to ensure complete dissolving of FeCl_3_. Then 3.6 g of NaOAc was added to the clear solution to dissolve it completely. Afterward, 0.65 g of NAC was added to the solution and again stirred thoroughly until completely dissolved. Finally, the clarified mixed solution was transferred to the reaction kettle and reacted at 200 °C for 12 h in the incubator. After natural cooling, greigite nanozyme was washed with ethanol and double distilled water respectively three times, and then lyophilized in a freeze dryer.

Morphological of greigite nanozyme was determined with a scanning electron microscope (SEM, S‐4800II, Hitachi) and transmission electron microscope (TEM, Tecnai 12, Philips). Powder X‐ray diffraction (XRD) patterns of the greigite nanozyme were obtained using a D8 Advance polycrystalline X‐ray diffractometer XRD (D8 Advance, Bruker AXS). X‐ray photoelectron spectroscopy (XPS) of greigite nanozyme was measured on an ESCALAB 250Xi X‐ray photoelectron spectrometer (ESCALAB 250Xi, Thermo Scientific) with a monochromatic Al K*α* source. All XPS peaks were calibrated using C1s (284.8 eV) as the reference. Zeta potentials for greigite nanozyme suspensions were determined by a Malvern ZEN3690 Zeta sizer (Malvern, UK).

### SOD, CAT, ^•^OH Scavenging Activity, GPX‐Like Activity, and TEAC of Greigite Nanozyme

${}^{•}O_{2}^{-}$ The superoxide anion (•O_2_^−^) scavenging activity of greigite nanozyme was assessed using SOD enzyme activity kit. The principle of detection was that the reaction system of xanthine and xanthine oxidase produced superoxide anion (•O_2_^−^), which could reduce nitrogen blue tetrazolium (NBT) to blue formazan, and the latter had strong absorption at 560 nm. Superoxide dismutase could remove •O_2_^−^ and inhibit the formation of formazan. The lighter the blue of the reaction solution, the higher the SOD activity.

Catalase can decompose H_2_O_2_ into H_2_O and O_2_. The catalase activity of greigite nanozyme was tested by measuring the dissolved oxygen content in the solution using a dissolved oxygen meter (Lightning Magnetic JPSJ‐606L).

The ^•^OH scavenging activity of greigite nanozyme was assessed using ^•^OH scavenging ability detection kit. According to the reagent description, H_2_O_2_/Fe^2+^ produces ^•^OH via the Fenton reaction, which oxidizes Fe^2+^ to Fe^3+^ in the ferroin aqueous solution, resulting in a decrease in the absorbance value at 536 nm. The ability of the greigite nanozyme to scavenge ^•^OH was calculated based on the degree of inhibition of the rate of decrease in absorbance for each group.

The GPx‐like activity of greigite nanozyme was estimated by the total glutathione detection testing kit. This kit utilized an indirect method of measurement. Glutathione peroxidase (GPx) catalyzed the production of GSSG from GSH, while glutathione reductase catalyzed the production of GSH from GSSG using NADPH, and the level of glutathione peroxidase activity could be calculated by measuring the reduction in NADPH.

The total antioxidant capacity of greigite nanozyme was estimated by total antioxidant capacity detection kit. This kit used 2,2'‐azino‐bis (3‐ethylbenzthiazoline‐6‐sulfonic acid) (ABTS) as a chromogenic agent, which could be oxidized to green ABTS^+^ under the action of appropriate oxidants, and the production of ABTS^+^ would be inhibited in the presence of antioxidants. The total antioxidant capacity of the sample could be determined and calculated by measuring the absorbance of ABTS^+^ at 734 nm or 405 nm.

### Tissue Specimens

With informed consent, eight degenerative nucleus pulposus tissues were surgically obtained from patients with lumbar disc herniation. In this study, patients collected during the operation did not have other complications such as osteoporosis. The collection and use of the nuclear tissue specimens were approved by the Ethical Committee of Medical College, Yangzhou University (approval number: YXYLL‐2020‐289).

### Isolation, Culture, and Identification of Rat NPCs

To isolate rat NP cells, ten SD male rats (8 weeks old) were euthanized by an overdose of pentobarbital sodium. The NP tissue of the rat tail IVD was extracted on a sterile operating table and digested with 0.5% collagenase type II for 12 h at 37 °C. Then the suspension was centrifuged at 1000 rpm for 3 min and the precipitate was incubated using 10% fetal bovine serum in DMEM/F‐12. With 5% CO_2_ at 37 °C, cells were digested and transferred into a sterile flask at a proper density. The medium was renewed every other day, and the first three generations of NPCs were used in the experiments. Toluidine blue staining and Aggrecan immunofluorescence staining were used to identify nucleus pulposus cells.

### Cell Viability Assay

In vitro cytotoxicity was detected by CCK‐8 assay. NPCs were seeded at the density of 5000 cells per well in 96‐well plates and incubated at 37 °C for 24 h under 5% CO_2_. Then cells were treated with different concentrations of greigite nanozyme and H_2_O_2_ for 24 h and the cell viability was detected by CCK‐8 assay. For the greigite nanozyme‐mediated protection of H_2_O_2_ stimulated cells experiment, NPCs were seeded at the density of 5000 cells per well in 96‐well plates and incubated at 37 °C for 24 h under 5% CO_2_. The next day, cells were treated with different concentrations of greigite nanozyme for 1 h, then cocultured with H_2_O_2_ (100 µm) for 24 h and finally the cell viability was detected by CCK‐8 assay.

### Reactive Oxygen Species Detection

The level of ROS in vitro was detected by a Reactive Oxygen Species Assay Kit. The rat NPCs (2 × 10^4^ cells per well in 12‐well plate) first incubated with greigite nanozyme or its supernatant for 1 h were next cocultured with H_2_O_2_ (100 µm) or PBS for 24 h. Then the cells were washed three times with PBS before loading the DCFH‐DA and DHE fluorescence probe for 20 min at 37 °C. Finally, cells were washed three times by PBS and imaged by a fluorescence microscope. The MDA content was assayed with a Lipid Peroxidation MDA Assay Kit after the same cell treatment as above following the manufacturer's instructions.

### Mito‐Tracker Red CMXRos Staining

The fluorescent probe Mito‐Tracker Red CMXRos (Beyotime, China) was used to determine the mitochondrial membrane potential (MMP). NPCs incubated with greigite nanozyme or the supernatant for 1 h were next cocultured with H_2_O_2_ (100 µm) or PBS for 24 h. Next, the cells were gently rinsed thrice with PBS and incubated with CMXRos (100 nm) at 37 °C for 20 min. Then, DAPI staining was performed after 4% paraformaldehyde fixation and 0.2% Triton X‐100 permeability. Finally, the cells were observed under a fluorescence microscope (ZEISS).

### Senescence‐Associated *β*‐Galactosidase Staining

The NPCs in the 6‐well plate were washed twice with PBS after the aforementioned culture method of greigite nanozyme and H_2_O_2_. According to the manufacturer's instructions, SA‐*β*‐Gal staining fixation solution was added to each well after fixed for 15 min at room temperature. The 6‐well plate was sealed with parafilm and then incubated at 37 °C overnight. Finally, the senescence expression of NPCs was observed using a microscope.

### Cellular Immunofluorescence Staining

The NPCs were seeded in a 24‐well plate (2 × 10^4^ cells per well), and different interventions were performed as aforementioned. Then cells were fixed with 4% paraformaldehyde for 20 min. After washing three times with PBS, cells were blocked at room temperature for 2 h. Then 250 µL of the following corresponding primary antibodies were added to each well: Collagenase type II (1:100), Aggrecan (1:400), TNF‐*α* (1:200), IL‐1*β* (1:200), p21Waf1/Cip1 (1:200), MMP9 (1:200), MMP13 (1:200). The samples were then incubated overnight at 4 °C. The next day, the samples were rinsed three times with PBS, and the cells were treated with the corresponding fluorescent secondary antibody (Goat Anti‐Mouse IgG H&L, Goat Anti‐Rabbit IgG H&L) at 37 °C for 2 h. Then the cells were incubated with DAPI for 3 min. Photographs were taken continuously with fluorescence microscope.

### Real‐Time Quantitative Polymerase Chain Reaction (RT‐qPCR)

Total RNA was extracted from NPC with Trizol Reagent (Vazyme) and then concentrations were determined using a DNA/Proteins Analyzer P100/P100+(Pultton). Next, 1 µg of total RNA was used for reverse transcription to synthesize complementary DNA (cDNA) with PrimeScript RT Master Mix (Vazyme), following the manufacturer's explanatory memorandum. Real‐time PCR Mix (TaKaRa, Shiga, Japan) was used on a light cycler (Roche, Basel, Switzerland) for RT‐PCR, with primers as shown in Table S1, Supporting Information.

### Animals Grouping and Surgical Procedures

All the animals were housed and treated by following the Ethical Committee of Yangzhou University (approval number: YXYLL‐2020‐289). Forty rats were randomly divided into four groups. Before surgical procedures, rats were anesthetized by intraperitoneal injection of pentobarbital sodium. The tail was sterilized with iodinated polyvinylpyrrolidone and then the disc was punctured with a 21G needle between the 6th to 7th and 7th to 8th caudal vertebra (Co6‐8). After the puncture was completed, 10 µL of the following solutions were injected into the rat intervertebral disc with a microinjector: control group (no treatment), model group (PBS), supernatant group (greigite nanozyme supernatant, 20 µg mL^−1^), and greigite nanozyme group (greigite nanozyme solution, 20 µg mL^−1^). After that, the mice were disinfected with iodine volts again and placed in cages with regular changes of feed, bedding, and water.

### Radiographic and MRI Analysis

Four weeks after puncture surgery, each group of rats was randomly selected to undergo X‐ray and MRI scans before sacrifice. Rats were kept in the supine position with their tails extended and placed on the molybdenum target radiographic‐image unit (GE SIGNA Architect). MRI coronal t2‐weighted images were taken to assess signal and structural differences in the intervertebral disc. The IVD height and the adjacent upper and lower vertebral body heights were measured using image measurement tools, and the disc height index (DHI) was calculated from these values.

### Tissue Section Staining (HE and Safranin‐O Staining)

Rats were sacrificed with intraperitoneal injection of an overdose of pentobarbital sodium, and then tail vertebral specimens were collected. Specimens were fixed in 10% formalin solution for 48 h, soaked in 10% EDTA until complete decalcification, dehydrated, and finally embedded in paraffin. The intervertebral discs were cut into 8 µm sections. In order to acquire histological evaluation, paraffin tissue sections were stained with hematoxylin‐eosin (HE), safranin o‐fast green, and alcian blue according to the instructions for use of the corresponding reagents.

### Statistical Analyses

All data are presented as the mean values ± standard deviations (SD) and were analyzed using the GraphPadPrism9 software (GraphPad Software, USA). Comparisons of various treatment groups were performed using analysis of variance (ANOVA) with Tukey's post hoc test. *p* < 0:05 was considered statistically significant.

## Conflict of Interest

The authors declare no conflict of interest.

## Author Contributions

Y.S. and H.L. contributed equally to this article. H.C. and Z.X. designed the experiments. W.L. constructed the animal models and collected the samples. Y.S., H.L., and K.L. conducted the experiments and analyzed the data. H.W. and D.L. supervised the performace of experiments. Y.S. and H.C. wrote the manuscript. H.C., B.L., L.G., and Z.X. revised the manuscript. B.L. and L.G. were consulted during the whole study process. All authors have approved the final version of the manuscript.

## Supporting information

Supporting Information

## Data Availability

The data that support the findings of this study are available from the corresponding author upon reasonable request.
