# Supplementary material for: Rescuing Nucleus Pulposus Cells From Senescence via Dual‐Functional Greigite Nanozyme to Alleviate Intervertebral Disc Degeneration
Source: Adv Sci (Weinh). 2023 Jul 3;10(25):2300988. doi: 10.1002/advs.202300988 (PMC10477883; doi:10.1002/advs.202300988)
Supplement: Supplementary file 1 — Supporting Information [file ADVS-10-2300988-s001.pdf]

ADVANCED SCIENCE
Open Access

Supporting Information

for *Adv. Sci.*, DOI 10.1002/advs.202300988

Rescuing Nucleus Pulposus Cells From Senescence via Dual-Functional Greigite Nanozyme to Alleviate Intervertebral Disc Degeneration

Yu Shi, Hanwen Li, Dongchuan Chu, Wenzheng Lin, Xinglong Wang, Yin Wu, Ke Li, Huihui Wang, Dandan Li, Zhuobin Xu, Lizeng Gao*, Bin Li* and Hao Chen**

Rescuing nucleus pulposus cells from senescence via dual-functional greigite nanozyme to alleviate intervertebral disc degeneration

Yu Shi[1,2,#], Hanwen Li[3,#], Dongchuan Chu[4], Wenzheng Lin[1,2], Xinglong Wang[1,2], Yin Wu[1,2], Ke Li[1,2], Huihui Wang[2], Dandan Li[2], Zhuobin Xu[1,2,*], Lizeng Gao[5,*], Bin Li[3,*], Hao Chen[1,*]

1. Department of Orthopedics, Affiliated Hospital of Yangzhou University, Yangzhou, P.R. China.
2. Institute of Translational Medicine, Medical College, Yangzhou University, Yangzhou, P.R. China.
3. Orthopedic Institute, Department of Orthopedic Surgery, First Affiliated Hospital, Suzhou Medical College, Soochow University, Suzhou, P.R. China
4. Department of Radiology, Affiliated Hospital of Yangzhou University, Yangzhou, P.R. China.
5. CAS Engineering Laboratory for Nanozyme, Institute of Biophysics, Chinese Academy of Sciences, Beijing, P.R. China

# These authors contributed equally to this article.

* Corresponding author:

Hao Chen E-mail: hchen2020@yzu.edu.cn

Bin Li E-mail: binli@suda.edu.cn

Lizeng Gao Email: gaolizeng@ibp.ac.cn

Zhuobin Xu E-mail: xuzb@yzu.edu.cn

Supplementary Figures:

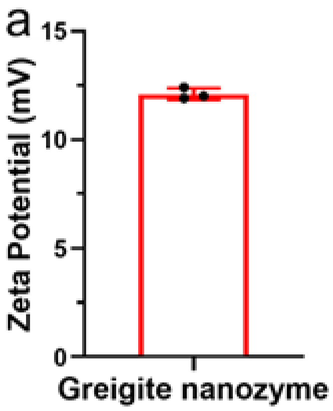

Figure S1 Zeta potential of greigite nanozyme.

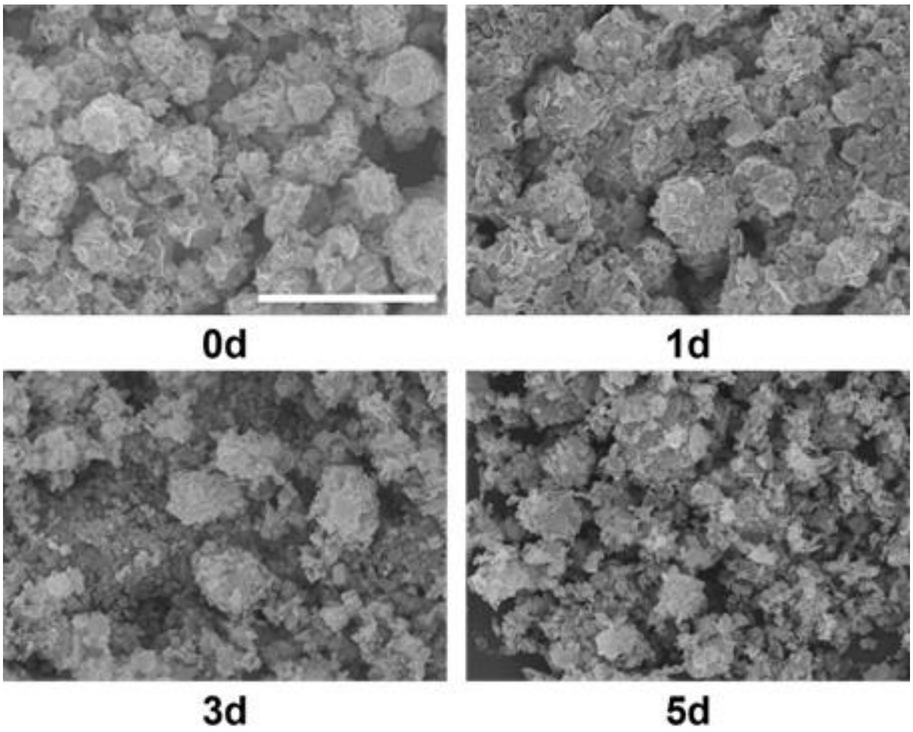

Figure S2 The degradation process of greigite nanozyme. SEM images of greigite nanozyme at different degradation times (scale bar = 10μm).

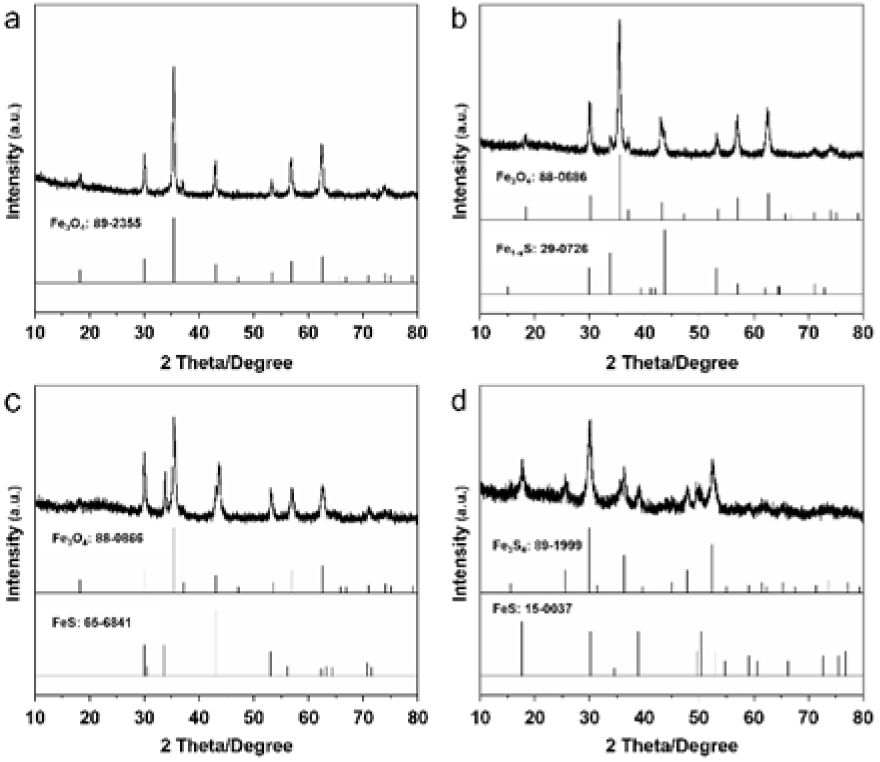

Figure S3 The XRD pattern of greigite nanozyme synthesized with different concentrations of NAC. The XRD pattern of greigite nanozyme synthesized with 0 mM NAC (a), 1mM NAC (b), 2 mM NAC (c) and 3mM NAC (d).

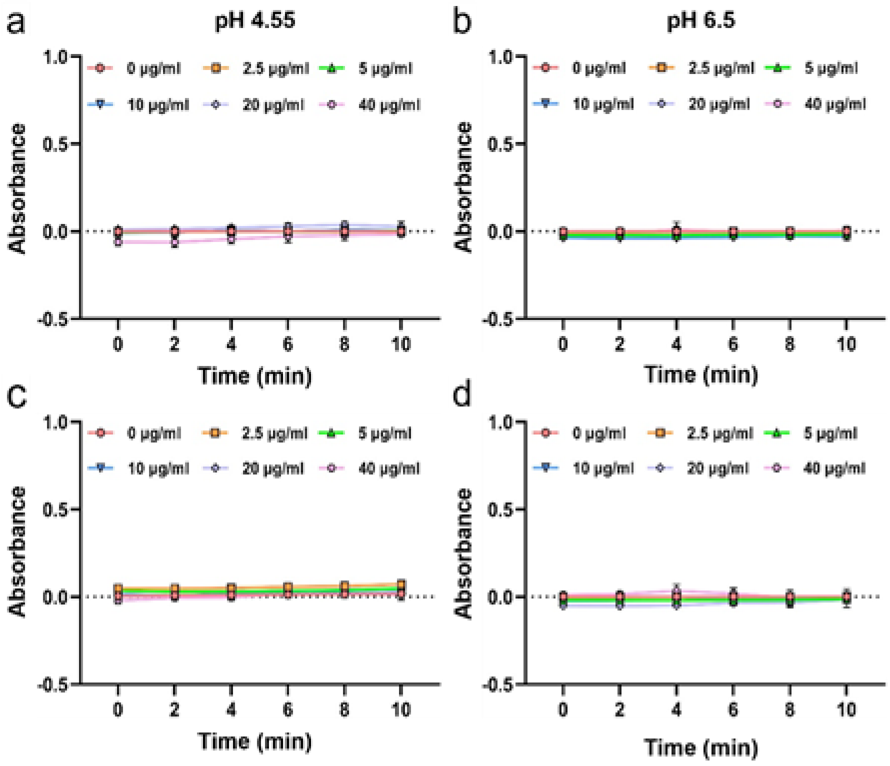

Figure S4 Determination of quasi-peroxidase activity and quasi-oxidase activity of greigite nanozyme at different pH. a) Quasi-oxidase activity of greigite nanozyme at pH4.5. b) Quasi-oxidase activity of greigite nanozyme at pH6.5. c) Quasi-peroxidase activityof greigite nanozyme at pH4.5. d) Quasi-peroxidase activity of greigite nanozyme at pH6.5.

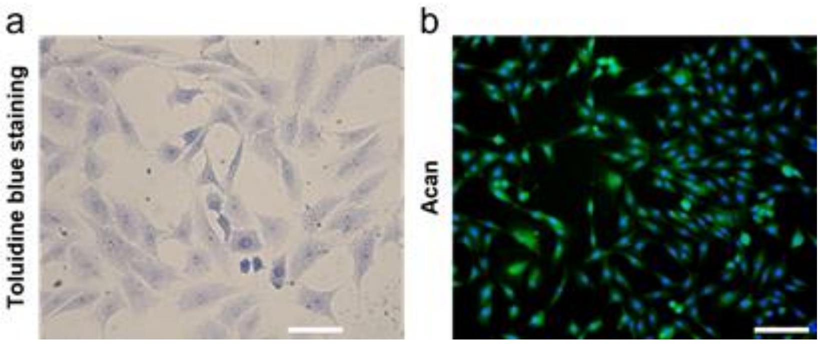

Figure S5 Identification of NPCs. a) Toluidine blue staining of NPCs (scale bar = 100 μm). b) Acan immunofluorescence staining of NPCs (scale bar = 100 μm).

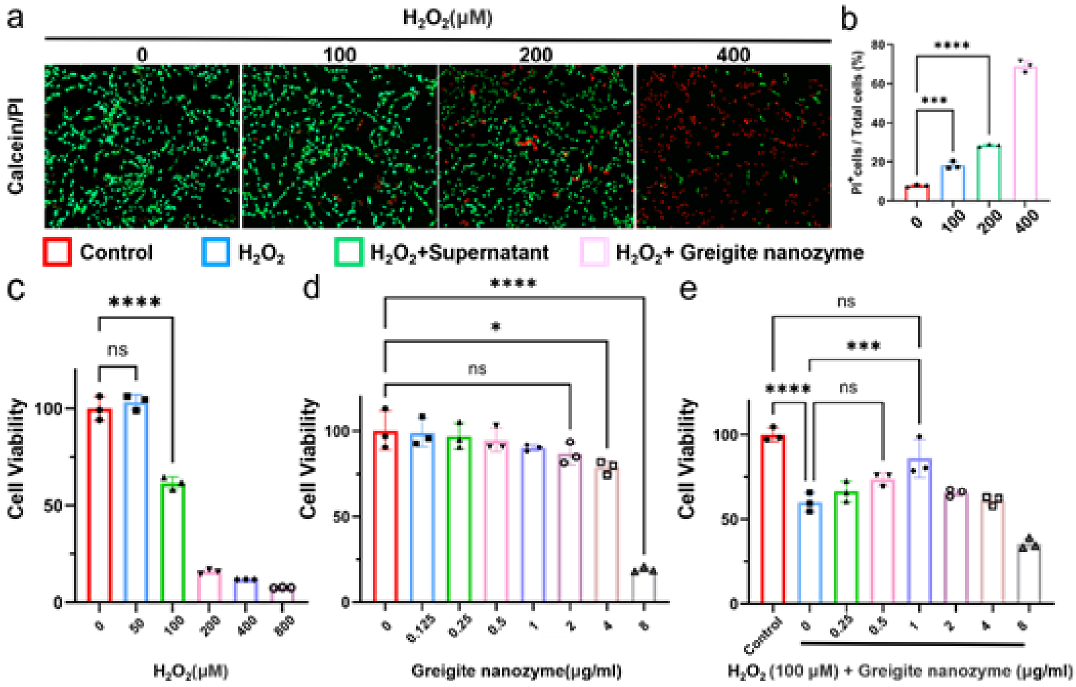

Figure S6 Biocompatibility of greigite nanozyme. a, b) The calcein/PI co-staining of NPCs cultured by different concentration gradient H_2O_2 and semi-quantitative analysis of fluorescence intensity (scale bar = 200 μm). c) Effect of different concentration gradient H_2O_2 on viability of NPCs. d) Biotoxicity of greigite nanozyme in NPCs. e) Protective effect of greigite nanozyme on NPCs in oxidative stress microenvironment. (* means *P* value<0.05, *** means *P* value<0.001, **** means *P* value<0.0001)

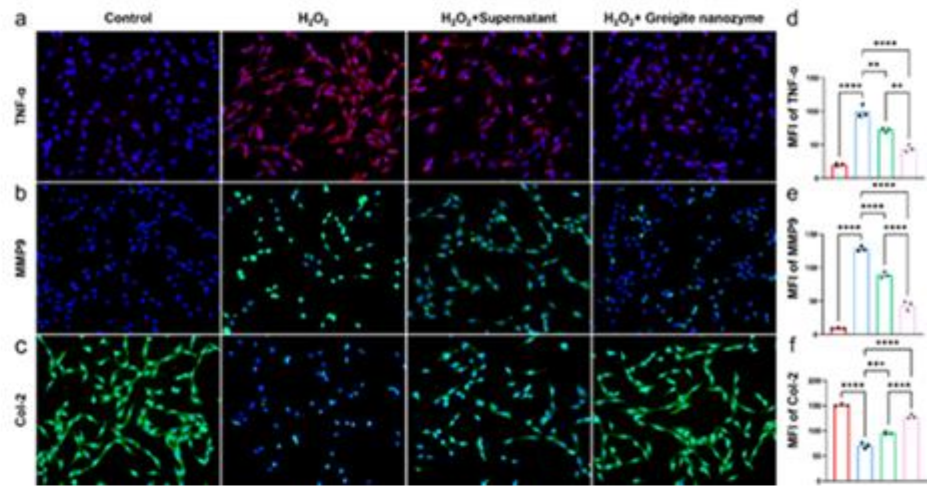

Figure S7 Anti-inflammation, anti-catabolism and anabolism-promoting effects of greigite nanozyme on NPCs. a) Immunofluorescence staining results of TNF-α in different groups (scale bar = 100 μm). b) Immunofluorescence staining results of MMP9 in different groups (scale bar = 100 μm). c) Immunofluorescence staining results of Col-2 in different groups (scale bar = 100 μm). d) Semi-quantitative analysis of fluorescence intensity of TNF-α. e) Semi-quantitative analysis of fluorescence intensity of MMP9. f) Semi-quantitative analysis of fluorescence intensity of Col-2. (** means P value<0.01, *** means P value<0.001, **** means P value<0.0001)

Supplementary Table:

Gene	Forward primers	Reverse primers
GAPDH	GCAAGTTCAACGGCACAG	CGCCAGTAGACTCCACGAC
Aggrecan	CGCTACTCGCTGACCTTT	GCTCATAGCCTGCTTCGT
MMP-3	TTTGGCCGTCTCTTCCATCC	GCATCGATCTTCTGGACGGT
TNF-α	TACTGAACTTCGGGGTGATTGGTCC	CAGCCTTGTCCCTTGAAGAGAACC
IL-1β	GACTTCACCATGGAACCCGT	GGAGACTGCCCATTCTCGAC
ADAMTS-5	GGACCTACCACGAAAGCAGATC	GCCGGGACACACGGAGTA

Table S1 Primers used for RT-qPCR in the study
